# DeepCTG® 1.0: an interpretable model to detect fetal hypoxia from cardiotocography data during labor and delivery

**DOI:** 10.3389/fped.2023.1190441

**Published:** 2023-06-15

**Authors:** Imane Ben M’Barek, Grégoire Jauvion, Juliette Vitrou, Emilia Holmström, Martin Koskas, Pierre-François Ceccaldi

**Affiliations:** ^1^ Department of Gynecology Obstetrics, Assistance Publique des Hôpitaux de Paris -Beaujon, Clichy, France; ^2^ Health Simulation Department, iLumens, Université Paris Cité, Paris, France; ^3^ R&D Department, Genos Care, Paris, France; ^4^ Department of Gynecology-Obstetrics and Reproduction, Assistance Publique des Hôpitaux de Paris -Bichat, Paris, France; ^5^ Department of Gynecology-Obstetrics and Reproduction, Hôpital Foch, Suresnes, France

**Keywords:** cardiotocography, computerized cardiotocography, fetal monitoring, fetal hypoxia, labor, Fetal Heart Rate (FHR), fetal morbidity, fetal mortality

## Abstract

**Introduction:**

Cardiotocography, which consists in monitoring the fetal heart rate as well as uterine activity, is widely used in clinical practice to assess fetal wellbeing during labor and delivery in order to detect fetal hypoxia and intervene before permanent damage to the fetus. We present DeepCTG® 1.0, a model able to predict fetal acidosis from the cardiotocography signals.

**Materials and methods:**

DeepCTG® 1.0 is based on a logistic regression model fed with four features extracted from the last available 30 min segment of cardiotocography signals: the minimum and maximum values of the fetal heart rate baseline, and the area covered by accelerations and decelerations. Those four features have been selected among a larger set of 25 features. The model has been trained and evaluated on three datasets: the open CTU-UHB dataset, the SPaM dataset and a dataset built in hospital Beaujon (Clichy, France). Its performance has been compared with other published models and with nine obstetricians who have annotated the CTU-UHB cases. We have also evaluated the impact of two key factors on the performance of the model: the inclusion of cesareans in the datasets and the length of the cardiotocography segment used to compute the features fed to the model.

**Results:**

The AUC of the model is 0.74 on the CTU-UHB and Beaujon datasets, and between 0.77 and 0.87 on the SPaM dataset. It achieves a much lower false positive rate (12% vs. 25%) than the most frequent annotation among the nine obstetricians for the same sensitivity (45%). The performance of the model is slightly lower on the cesarean cases only (AUC = 0.74 vs. 0.76) and feeding the model with shorter CTG segments leads to a significant decrease in its performance (AUC = 0.68 with 10 min segments).

**Discussion:**

Although being relatively simple, DeepCTG® 1.0 reaches a good performance: it compares very favorably to clinical practice and performs slightly better than other published models based on similar approaches. It has the important characteristic of being interpretable, as the four features it is based on are known and understood by practitioners. The model could be improved further by integrating maternofetal clinical factors, using more advanced machine learning or deep learning approaches and having a more robust evaluation of the model based on a larger dataset with more pathological cases and covering more maternity centers.

## 1. Introduction

Cardiotocography (CTG) is defined as the recording of Fetal Heart Rate (FHR) and Uterine Contractions (UC) during pregnancy using an electronic fetal monitor. It is used during labor and delivery as a screening tool to monitor the fetal well-being.

Currently, its interpretation is mainly performed visually by obstetricians or midwives following common guidelines (1). Although the guidelines are constantly being challenged (2, 3), CTG interpretation methods have not drastically changed since CTG was introduced as a screening tool in the 1960s. The overall process of interpreting CTG during delivery is known to be subject to a significant inter-observer and intra-observer variability (4, 5). For those reasons, the effectiveness of continuous CTG during labor is still debated (6). Some professionals recommend fetal blood sampling when there are concerns about abnormal fetal heart rate patterns (7, 8). In addition to their questionable contribution to reducing poor neonatal outcomes, these invasive methods are not without risks for the fetus, can be difficult to perform and require available medical staff (9, 10). Therefore, it is worth improving computerized CTG analysis systems that may one day replace these invasive technologies.

In the last decades, researchers worked on designing reliable computer-aided systems able to process automatically CTG signals to detect fetal hypoxia (11). The first developed systems were based on a quantitative adaptation of the guidelines proposed by the International Federation of Gynecology and Obstetrics (FIGO) (12). Dawes and Redman have designed a system in the 1980s to alert practitioners during pregnancy on the risk of pathological outcome (13). The SisPorto system, developed by Ayres-de-Campos et al., consists of a quantitative adaptation of FIGO's guidelines. The last iteration of the system, SisPorto 4.0, has been released in 2015 (14). Georgieva et al. published in 2017 the OxSys system (15), based on the decelerative capacity, an average measure of the downward movements in the FHR signal. More recently, the construction of large clinical databases of CTG signals with corresponding clinical information and fetal outcomes (16, 17), some of whom are published in open-access, have led to a surge of research works about computerized CTG analysis using recent machine learning and deep learning techniques. Gatelier et al. and Abry et al. have trained and evaluated multivariate machine learning models on the open CTU-UHB dataset (18, 19). Other systems are based on deep learning methods which input the raw CTG signals instead of features extracted from the signals: Petrozziello et al. and Mohannad et al. have built such systems on proprietary databases with more than 30.000 births (20, 21), while Fergus et al. and Ogasawara et al. have built similar systems on the CTU-UHB dataset (22, 23).

In the present study, we introduce DeepCTG® 1.0, a model able to predict fetal acidosis during labor and delivery using CTG signals and evaluated on several clinical datasets.

## 2. Materials and methods

### 2.1. Datasets used

The model was built and evaluated using three datasets (Table 1):

– The CTU-UHB dataset (16) contains 552 open-access CTG recordings collected at the University Hospital of Brno, with corresponding maternofetal data (eg gestational age, mother's age, parity) and fetal outcome (fetal blood pH, Apgar at one and five minutes, weight). Those cases have been annotated by nine expert obstetricians who predicted the labor outcome (pH < 7.15) based on the CTG signals (5). We also have defined a college of obstetricians which consists in considering for every case the most frequent labor outcome prediction among the nine obstetricians. Some annotations are missing: the nine obstetricians have classified in average 456 cases each over 552.
– The SPaM dataset, introduced as part of the Workshop on Signal Processing and Monitoring in Labor (24), contains 300 cases collected from three participating centers (Lyon, Brno and Oxford). Each center provided 100 cases: 80 with a fetal pH within 7.25–7.30 and 20 corresponding to a pathological outcome (pH < 7.05). For every case, the CTG recordings during delivery (FHR and UC) as well as the binary outcome (pH < 7.05 or pH within 7.25–7.30) are available.
– The Beaujon dataset contains 675 CTG recordings collected at the University Hospital Beaujon (Clichy, France), with corresponding maternofetal data and fetal outcomes. All deliveries with pH < 7.15 between January 2020 and December 2022 have been included, and for any such delivery, the latest delivery with pH > 7.15 has been included. This inclusion methodology has been defined to limit the total number of cases (because the extraction of the CTG signals was manual and time-consuming) while ensuring that the dataset contains the highest possible number of cases of fetal hypoxia.

**Table 1:** Datasets’ characteristics.

	**CTU-UHB**	**Beaujon**	**SPaM**
Total number of cases	552	675	300
**Fetal outcome**			
pH < 7.05 (Nb)	40	42	60
7.05 ≤ pH < 7.15 (Nb)	65	257	240
pH ≥ 7.15 (Nb)	447	376	
Apgar 1': mean (std)	8.3 (1.6)	8.4 (2.5)	–
Apgar 1': Nb < 7	68	140	
Apgar 5': mean (std)	9.1 (1.1)	9.4 (1.5)	
Apgar 5': Nb < 7	19	43	
Term: mean (std)	40.0 (1.1)	39.6 (1.5)	
Birth weight: mean (std)	3.4 (0.5)	3.3 (0.5)	
Sex (share of girls)	48%	46%	
**Maternal and delivery data**			
Maternal age: mean (std)	29.7 (4.5)	29.7 (5.1)	–
Share of primiparous	68%	56%	
Delivery (share of cesareans)	8%	12%	
**CTG signals**			
Mean signal duration (min)	74	420	308
Share of FHR missing points	19%	7%	7%
Share of UC missing points	20%	22%	5%

An additional dataset shared by Boudet et al. (25), named the FHRMA dataset (for FHR morphological analysis) in the rest of the paper, has been used to calibrate our FHR baseline estimation methodology. It contains, for 66 FHR recordings, a reference baseline, accelerations and decelerations annotated by a consensus of four obstetricians.

### 2.2. Signals preprocessing and filtering

The CTG signals fed to the model correspond to the latest available data before delivery. The signals are available on a 4 Hz frequency. There are a significant proportion of missing points in the signals, equal to 0 or −1 in the raw signals (between 5% and 20% depending on the datasets), and they were preprocessed using the following approach:

– Missing segments of data lasting less than 10 min are interpolated using linear interpolation. Missing segments lasting more than 10 min are not filled.
– For every case, the latest 30 min segment without any missing data (after interpolation) is selected. If such a segment does not exist, or if it starts more than 90 min before delivery, the case is discarded. This led to the discarding of 10 cases over the three datasets (including 1 pathological case), representing less than 0.7% of the total number of cases.

Some existing studies perform missing data imputation using linear interpolation (21, 26), and some other use more advanced interpolation methods like spline interpolation (22, 27) or autoregressive models (28). We have implemented and tested a more advanced gap imputation technique based on cubic Hermite spline interpolation and used in another paper (22), using the python function scipy.interpolate.CubicHermiteSpline (https://docs.scipy.org/doc/scipy/reference/generated/scipy.interpolate.CubicHermiteSpline.html), but it did not improve the performance of the model. We have not found other studies that limit the maximum duration to fill the data gaps: choosing 10 min helped to limit the size of the interpolated intervals while discarding a limited number of cases.

### 2.3. Features computed from the CTG signals

**Figure 1:**
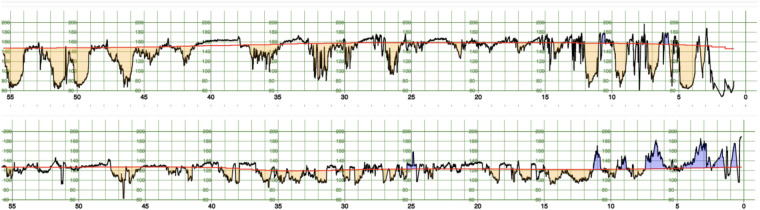
FHR signal (black) for two cases of the CTU-UHB dataset, one case with a pathological outcome (pH < 7.05, top) and one with a normal outcome (bottom). The baseline is displayed in red, accelerations in blue and decelerations in orange.

This section details the computation of the 25 features extracted from the CTG signals which have been considered in the prediction model. For illustration, Figure 1 shows the raw FHR signals with the corresponding baseline, accelerations and decelerations determined as described in the paper.

#### 2.3.1. Features based on FHR baseline

Three features are computed from the FHR baseline: the minimum, median and maximum values computed on the 30 min segment fed to the model.

The FHR baseline is defined as the mean of the signal after accelerations and decelerations have been excluded. This creates a circular definition as accelerations and decelerations are defined as periods when the signal is consistently above or below the baseline. Because of this circular definition and of the high variability of some recordings which makes hard to distinguish between a change in the baseline and an acceleration or a deceleration, designing a methodology to automatically compute the FHR baseline from the CTG signal is a complex and ill-defined problem. To overcome this issue, we calibrate our methodology for baseline estimation on the baselines, accelerations and decelerations annotated by a consensus of obstetricians available in the FHRMA dataset.

Houzé de l'Aulnoit et al. have implemented and compared 11 published baseline estimation methods with baselines annotated by a consensus of four obstetricians in the FHRMA dataset (29). The same team has developed a methodology based on a weighted median filter and has shown that it outperforms other existing approaches on the FHRMA dataset (30). We compute the FHR baseline with a similar weighted median filter relying on the following methodology:

– The FHR signal is supposed at any time to be in one of two states: a non-stable state, corresponding to accelerations and decelerations, and a stable state corresponding to the remaining time periods.
– The probability $p_{stab}(t)$ for the signal to be in a stable state at time *t* is modeled using a prediction model trained on the annotations available in the FHRMA dataset.
– Then, the FHR baseline at time *t* is estimated as a weighted median of the signal around time *t*, with weights depending on the $p_{stab}$ function.

The rest of this section details the estimation of $p_{stab}$ and the definition of the weights.

##### 2.3.1.1. Estimation of the probability of stability

We note $FHR_{a-b}$ the bandpass filtered FHR signal with cut-off frequencies *a* and *b* expressed in beats per minute (bpm), which corresponds to the FHR signal where only frequencies within a certain range are kept. Computing $FHR_{a-b}$ with different ranges of frequencies enables to isolate specific features observed at different frequencies in the FHR signal. We note d(X) the first derivative of a signal *X*, estimated by a simple finite differences algorithm, and envelope(X) the analytical envelope of signal *X*, which captures the slowly varying features of a signal *X*. In practice, $FHR_{a-b}$ is computed with a Butterworth sixth-order filter using the python function scipy.signal.butter (https://docs.scipy.org/doc/scipy/reference/generated/scipy.signal.butter.html), and envelope(X) is computed using the python function scipy.signal.hilbert (https://docs.scipy.org/doc/scipy/reference/generated/scipy.signal.hilbert.html).

The probability of stability $p_{stab}(t)$ is estimated using a logistic regression fed with the eight following variables:

$$\begin{array}{rl} | & d(FHR_{0\;bpm-0.1\;bpm})|,\;|d(FHR_{0.1\;bpm-1\;bpm})|,\;|d(FHR_{1\;bpm-3\;bpm})|,\;\\ & \left|d(FHR_{3\;bpm-7\;bpm})\right| \end{array}$$

$$\begin{array}{rl} | & envelope(d(FHR_{0\;bpm-0.1\;bpm}))|,\;|envelope(d(FHR_{0.1\;bpm-1\;bpm}))|,\\ & \;\;|envelope(d(FHR_{1\;bpm-3\;bpm}))|,\;|envelope(d(FHR_{3\;bpm-7\;bpm}))| \end{array}$$

Smoothing the signal with several intervals of frequencies (0–0.1 bpm, 0.1–1 bpm, 1–3 bpm, 3–7 bpm) enables to model the signal variability at a large range of frequencies and to adapt to the diversity of accelerations and decelerations observed in CTG signals. Those ranges of frequencies capture the observed variability of accelerations and decelerations, and we use four ranges of frequencies only to limit the number of variables fed to the logistic regression model estimating $p_{stab}(t)$. The absolute value of the first derivative of $FHR_{a-b}$ is used because points with a high derivative are likely to correspond to the slope of an acceleration or deceleration. However, using the first derivative only is not enough because it does not enable to distinguish between the baseline and the trough of an acceleration or deceleration. That is why we use the envelope of the derivative as well, that has a high value throughout the acceleration or deceleration. More details on the motivations for using those variables to model the stability of the signal are given in Boudet et al. (30).

The 66 recordings in the FHRMA dataset have been randomly separated into two datasets: a training dataset with 40 recordings used to train the logistic regression, and a test dataset with 26 recordings to evaluate its accuracy. For every recording, it is assumed that time periods annotated by the expert consensus as an acceleration or deceleration correspond to a non-stable state, while the remaining time periods correspond to a stable state.

##### 2.3.1.2. Weights used to compute the weighted median

The baseline at time *t* is computed as a weighted median of the FHR signal on a symmetric window centered on time *t* and of half-duration *T*. We note $W_{t}(t^{\prime })$ the weight associated to a time $t^{\prime }$ when estimating the baseline at time *t*. It is given by the following formula:

$$W_{t}(t^{\prime })=p_{stab}(t^{\prime })*\max\left(0,\;1-\frac{\left|t-t^{\prime }\right|}{T}\right)$$

The first term $p_{stab}(t^{\prime })$ ensures that time periods corresponding to accelerations and decelerations (where we should have $p_{stab}(t^{\prime })∼0$) are excluded from the computation of the baseline. The second term ensures that more weight is given to times $t^{\prime }$ close to the current time t: it equals 1 when $t=t^{\prime }$ and decreases to 0 when $|t-t^{\prime }|=T$.

We consider two different methodologies for setting the value of the half-duration of the window T:

– Fixed half-duration: in this configuration we set T=20minutes.
– Variable half-duration: in this configuration the half-duration of the window used to compute the baseline at any time *t* is modulated with $\bar{\;p_{stab}}(t)$, the average probability of stability in the interval [t−20minutes,t+20minutes]. It is given by the formula $T=\frac{20\;minutes}{3\;*\;\bar{\;p_{stab}}(t)}$. The factor 3 in front of $\bar{\;p_{stab}}(t)$ ensures that the average half-duration over all recordings remains close to 20minutes. The motivation for introducing a variable half-duration is that when the signal has a low stability ($\bar{\;p_{stab}}(t)$ is low), a larger window is necessary to be able to estimate accurately the baseline, while when the signal is stable ($\bar{\;p_{stab}}(t)$ is high), a smaller window is enough to estimate the baseline accurately and enables to adapt more quickly to a change in the baseline.

More details about the motivations for those weights are given in Boudet et al. (30), except the variable half-duration of the window which is introduced in our paper.

#### 2.3.2. Features based on FHR accelerations and decelerations

Accelerations and decelerations are defined in FIGO's guidelines (1) as increases in FHR above the baseline (respectively decreases in FHR below the baseline) of more than 15 bpms in amplitude and lasting more than 15 seconds. Consistently with this definition, a time period is defined to be an acceleration when the three following conditions are met:

– The FHR signal is always above the baseline during the time period.
– The maximum deviation to the baseline is higher than 15 bpms
– The average deviation to the baseline is higher than 10 bpms

We use a symmetric definition for decelerations. Then, 11 features are computed from the accelerations and decelerations detected in the 30 min segment:

– The number of accelerations, the total duration of the accelerations, the area covered by the accelerations (defined for every acceleration as the sum of the differences between the FHR and its baseline) and the maximum depth of the accelerations (defined as the maximum difference between the FHR and its baseline).
– The number of decelerations and late decelerations (lasting more than two minutes), the total duration of decelerations and late decelerations, the area covered by decelerations and late decelerations, and the maximum depth of the decelerations.

#### 2.3.3. Features based on the FHR variability

We compute two features based on the FHR variability and known to be linked to fetal hypoxia (18): the short-term variability (STV) and the long-term variability (LTV). There exist several slightly differing definitions and we have used the algorithm published by Dawes and Redman (31). We define STV as the difference in the mean FHR between two successive intervals lasting 3.75 s. LTV is defined as the amplitude (difference between maximum and minimum values) of the FHR signal in one-minute segments, as defined in FIGO's guidelines (1). STV and LTV computed on intervals are then averaged over the 30 min segment.

#### 2.3.4. Features based on contractions derived from UC signal

The contractions are identified from the UC signal as time periods during which the UC signal remains above 10 mm Hg during more than 30 s. Then, three features are defined: the number of contractions in the segment, the total duration of contractions and the area covered by contractions.

Decelerations and contractions are often interpreted jointly in clinical practice. The link between them is complex and is different if the deceleration is late or not (32). Consequently, we have defined several features based on both decelerations and contractions that match the joint interpretation of clinicians:

– The total duration of decelerations (or late decelerations) happening outside contractions and the area they cover.
– The time difference between the peaks of decelerations (or late decelerations) and the closest contraction peak, summed over the 30 min segment.

### 2.4. Training and evaluation of the model

The model is trained to predict a binary outcome corresponding to fetal hypoxia. The default outcome we use is pH < 7.05. The model is fed with a subset of the 25 features extracted from the CTG signals: several subsets of features are considered. It is trained using the python package scikit-learn (33). The cases are weighted to give the same total weight to cases with normal and pathological outcomes. This is required because the CTU-UHB and SPaM datasets are highly imbalanced and contain much more normal cases than pathological ones.

The main evaluation metric used is the area under the receiver operating characteristic curve (AUC), which is used in almost all papers evaluating fetal hypoxia prediction models. For every dataset, we estimate the performance of the model when it is trained on the other datasets, to ensure that the evaluation is representative of the performance that could be reached when using the model in a new center. On the CTU-UHB dataset, the performance of the model is compared with the nine expert obstetricians' annotations and with other published models, although this comparison is hard because other models are often evaluated on a different set of cases or with other outcomes. Also, we evaluate the impact of two key parameters on the performance of the model: the inclusion of cesareans in the dataset and the length of the CTG segment used to compute the features fed to the model. Those evaluations are performed on all datasets using cross-validation with five folds.

### 2.5. Interpretability of the model

The predicted risk of fetal hypoxia *p* can be written as $p=\frac{exp(\beta_{0}+\sum_{i}\beta_{i}x_{i})}{1+exp(\beta_{0}+\sum_{i}\beta_{i}x_{i})}$. where $x_{i}$ is a feature fed to the model and $\beta_{i}$ is the corresponding coefficient of the logistic regression. The contribution $c_{i}$ of every feature *i* in the risk of fetal hypoxia can be defined as $c_{i}=\frac{\beta_{i}x_{i}}{\sum_{i}\beta_{i}x_{i}\;}$. Several variations can be considered around this formula. For example, scaling and normalizing the values of each feature helps to produce interpretable contributions, and computing the contributions only on features with $\beta_{i}x_{i}>0$ enables to build contributions which are all positive and sum to 1.

Then, the contributions of each feature can be provided to practitioners along with the risk of fetal hypoxia. For example, an important contribution of the feature indicating a high value for the maximum FHR baseline may indicate a case of tachycardia.

### 2.6. Ethical approval

This work had the approval of Robert Debré hospital's Ethical committee (IRB 00006477).

## 3. Results

### 3.1. Missing data imputation method

We have evaluated the performance of the model with two different methodologies for missing data imputation: linear interpolation and cubic Hermite spline interpolation. This evaluation is performed on all datasets using a k-fold cross-validation strategy with five folds. Both models achieve very similar results (AUC = 0.760 with linear interpolation and AUC = 0.756 with cubic Hermite spline interpolation). This result is consistent with Asfaw et al. (34), which evaluates several data imputation techniques and concludes that using more advanced techniques than linear interpolation does not lead to a statistically significant improvement in the accuracy of the model.

We have also evaluated how decreasing the maximum duration to fill the data gaps from 10 min to 1 min impacted the performance of the model. Using 1 min led to the discarding of 275 cases, representing 22.4% of the total number of cases, including 36 pathological cases (24.8% of the total number of pathological cases) and 239 non-pathological cases (22.0% of the total number of non-pathological cases), and the AUC of the corresponding model was very similar (AUC = 0.764 with 1 min and AUC = 0.760 with 10 min), suggesting that including the cases with large data gaps does not significantly impact the accuracy of the model. Hence, we decided to use 10 min to ensure that our system applies to a larger share of the cases, even when the signal quality is particularly poor.

### 3.2. Accuracy of the baseline estimation

The accuracy of the baseline estimation is measured as the average difference between the baseline annotated by the consensus of obstetricians and the estimated baseline, over the 26 recordings in the FHRMA test dataset which have been excluded from the calibration of the baseline estimation.

Table 2 compares the accuracy of the estimated baseline in different settings:

– Baseline estimation with a constant probability of stability $p_{stab}=1$. In this setting, the baseline estimation is performed using a simple weighted median filter with weights depending on $\left|t-t^{\prime }\right|$ only.
– Baseline estimation with a probability of stability $p_{stab}$ calibrated on the FHRMA dataset and a fixed half-duration of the window.
– Baseline estimation with a probability of stability $p_{stab}$ calibrated on the FHRMA dataset and a variable half-duration of the window.

**Table 2:** Accuracy of the baseline estimation methodology on the FHRMA test dataset.

Baseline estimation methodology	Average difference with the baselines annotated by obstetricians (in beats per minute)
p_stab = 1	6.12
p_stab calibrated	5.03
p_stab calibrated and variable half-duration	4.75

Those results show that calibrating a probability of stability on the obstetricians' annotations significantly decreases the error (from 6.12 to 5.03 beats per minute), and that using a variable half-duration for the window leads to a significant decrease in the error as well (from 5.03 to 4.75 beats per minute).

### 3.3. Selection of the features fed to the model

#### 3.3.1. Univariate analysis

We have studied the individual performance of the 25 features extracted from the CTG signals by estimating a univariate logistic regression model for every one of those features. Table 3 shows the AUC of those univariate models as well as the *p*-value and the sign of the coefficient in the logistic regression. A positive sign means that the higher the variable the higher the risk of fetal hypoxia is. Those models are trained and evaluated on all available cases from the three datasets.

**Table 3:** Univariate models trained for the 25 features based on cardiotocography signals (all datasets).

	**AUC**	**Sign**	***p*-value**
**FHR baseline**			
Minimum value	0.604	-	<0.01
Median value	0.511	+	0.76
Maximum value	0.613	+	<0.01
**FHR accelerations and decelerations**			
Number of accelerations	0.605	+	<0.01
Total duration of accelerations	0.644	+	<0.01
Area of accelerations	0.649	+	<0.01
Maximum depth of accelerations	0.575	+	<0.01
Number of decelerations	0.562	+	<0.01
Number of late decelerations (>2 mn)	0.597	+	<0.01
Total duration of decelerations	0.661	+	<0.01
Total duration of late decelerations	0.609	+	<0.01
Area of decelerations	0.675	+	<0.01
Area of late decelerations	0.624	+	<0.01
Maximum depth of decelerations	0.551	+	0.02
**Uterine Contractions**			
Number of contractions	0.513	-	0.68
Total duration of contractions	0.517	+	0.58
Area of contractions	0.514	+	0.68
**Features based on contractions and FHR decelerations**			
Total duration of decelerations outside contractions	0.570	+	<0.01
Total duration of late decelerations outside contractions	0.583	+	<0.01
Area of decelerations outside contractions	0.588	+	<0.01
Area of late decelerations outside contractions	0.589	+	<0.01
Time difference between decelerations and contractions peaks	0.539	+	0.10
Time difference between late decelerations and contractions peaks	0.574	+	<0.01
**FHR variability**			
Short-term variability (STV)	0.582	+	<0.01
Long-term variability (LTV)	0.621	+	<0.01

The minimum and maximum value of the FHR baseline are both significant, unlike the median value. All features based on accelerations and decelerations are statistically significant, and the best performing ones in terms of AUC are the area covered by accelerations and the area covered by decelerations. The sign of the coefficients show that more accelerations or decelerations lead to a higher risk of fetal hypoxia. The features based on contractions are not statistically significant and do not have a great predictive power. The features based on a joint analysis of contractions and FHR decelerations perform better, however they are very correlated with the simpler features based on FHR decelerations only, and their predictive power is lower. Finally, the STV and LTV features both have a good predictive power, and high variabilities are associated to a higher risk of fetal hypoxia.

#### 3.3.2. Multivariate model selection

In a second step, several multivariate logistic regression models have been trained and evaluated on the three datasets using a k-fold cross-validation strategy with five folds. Table 4 gives the AUC of four multivariate models, both on the training and validation cases:

– A model fed with the 25 features computed from the CTG signals: this model is prone to overfitting and is hardly interpretable, but its performance on the training dataset can be interpreted as the maximum performance that can be achieved using any subset of those 25 features.
– A model fed with the best performing features in each category: the minimum and maximum value of the baseline (noted $b_{min}$ and $b_{max}$), the area covered by accelerations and decelerations (noted $acc_{area}$ and $dec_{area}$), the area covered by late decelerations outside contractions and the time difference between late decelerations and contraction peaks (noted $late\_dec\_no\_contraction_{area}$ and time_diff_late_dec_contraction), and the STV and LTV (noted stv and ltv). We have not included the features based on contractions which are not statistically significant.
– The same model after removing the features corresponding to the joint interpretation of contractions and decelerations.
– The same model after removing the STV and LTV features as well.

**Table 4:** Evaluation of several multivariate models (all datasets, k-fold cross-validation).

Features	AUC on training cases	AUC on validation cases
All (25 features)	0.793	0.750
b_min, b_max, acc_area, dec_area, stv, ltv, late_dec_no_contraction_area, time_diff_late_dec_contraction	0.773	0.747
b_min, b_max, acc_area, dec_area, stv, ltv	0.782	0.747
b_min, b_max, acc_area, dec_area	0.780	0.757

The results confirm that the model fed with the 25 features is the best performing one on the training cases (AUC = 0.793), however the evaluation on the validation cases leads to a significant decrease in performance (AUC = 0.750). The best performing model on the validation cases is the fourth one (AUC = 0.757), which is also the simplest as it is fed with four features only. In the rest of the paper, the prediction models are all fed with those four features: $b_{min}$, $b_{max}$, $acc_{area}$ and $dec_{area}$.

### 3.4. Evaluation of the model

**Figure 2:**
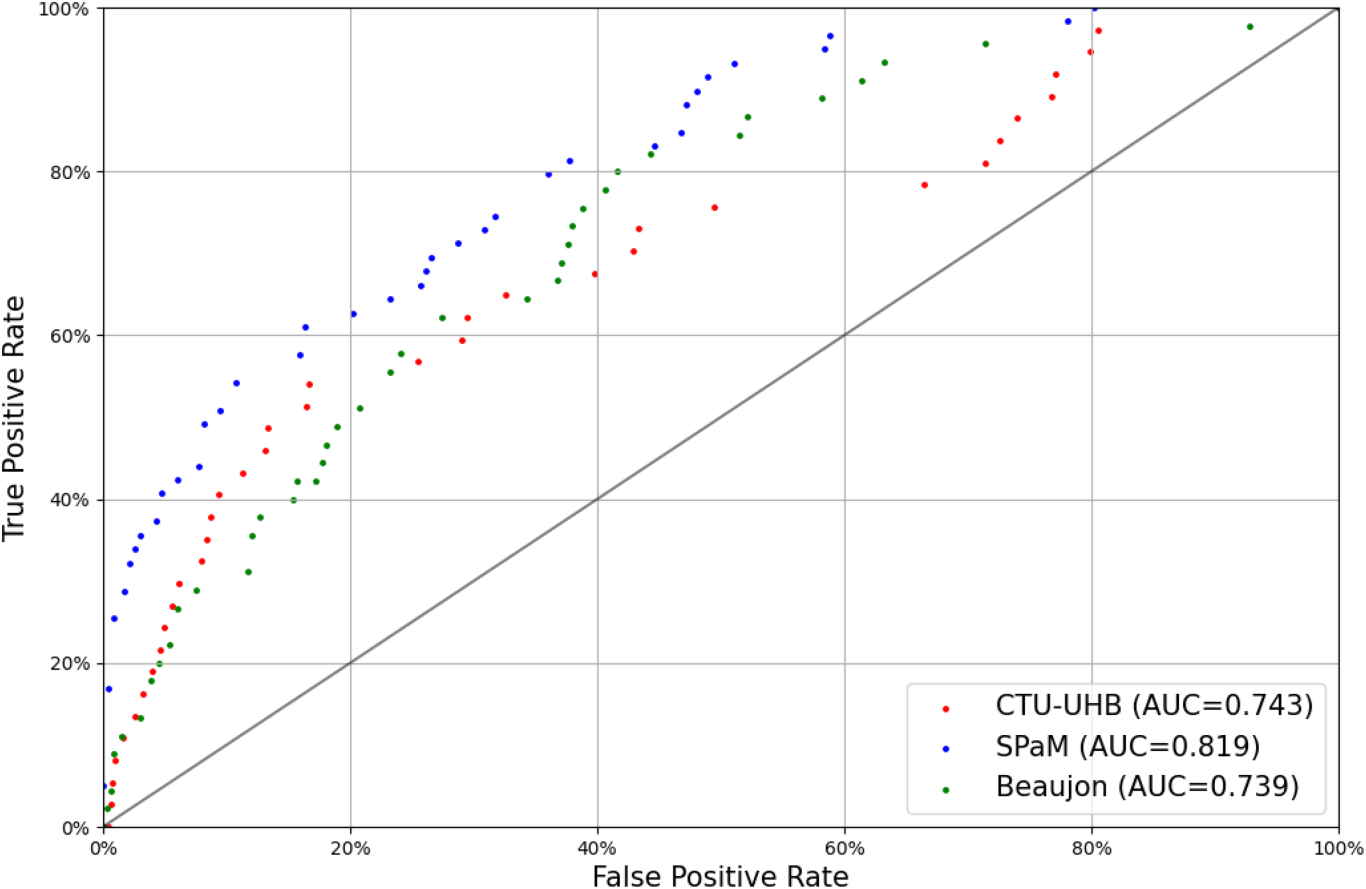
Receiver-operating characteristic curves of DeepCTG® 1.0 on the three datasets (outcome: pH < 7.05).

Table 5 shows, for each dataset, the AUC of the model when it is evaluated on the cases in this dataset and trained on the cases from the other datasets. For example, when evaluated on the CTU-UHB dataset, the model is trained on the Beaujon and SPaM datasets, and when evaluated on the Beaujon dataset, it is trained on the CTU-UHB and SPaM dataset. This methodology ensures that the evaluation is representative of the performance that could be reached when using the model in a new center. Figure 2 shows the corresponding ROC curves. The three centers forming the SPaM dataset have been grouped because the ROC curves on every center were too noisy due to the low number of cases of fetal hypoxia (20 in each center). The model reaches a similar performance on the CTU-UHB and Beaujon datasets (AUC = 0.743 and 0.739) and performs significantly better on the SPaM dataset (between 0.768 and 0.873).

**Table 5:** Evaluation and validation of DeepCTG® 1.0.

	**CTU-UHB**	**Beaujon**	SPaM		
			**Oxford**	**Lyon**	**Brno**
DeepCTG® 1.0 trained on other centers and evaluated on the center	0.743 (*)	0.739	0.811	0.873	0.768

* For example: DeepCTG® 1.0 trained on Beaujon, SPaM datasets and evaluated on CTU-UHB.

### 3.5. Comparison of the model with other published models

Table 6 compares the performance obtained by our model with other models published in the literature. All papers included in this comparison report the AUC obtained on the CTU-UHB dataset or a subset of it. For our model, we show a 95% confidence interval obtained by bootstrapping the CTU-UHB dataset with 100 bootstraps. A confidence interval was available in one other study only (23). We identify three main limitations to this comparison that are highlighted in Table 6:

– The outcomes predicted by the model are not the same in all papers: three papers use an outcome based on the fetal pH, one of them with a threshold different than 7.05, another paper uses an outcome based on both the fetal pH and the Apgar at one minute, and another one predicts the delivery mode (vaginal or caesarean).
– Three papers evaluate the model on a subset of the CTU-UHB cases. Fergus et al. (22) randomly select 101 cases to form the evaluation dataset, while Gatellier et al. (18) and Abry et al. (19) remove the cases with the most missing points in the FHR signals, which may bias the evaluation. For the two other papers, it is not explicitly stated whether the evaluation is performed on a subset of the cases or on all cases.
– Three papers train the model on the CTU-UHB dataset as well, which can significantly bias the performance evaluated on the same dataset.

**Table 6:** Comparison of DeepCTG® 1.0 with other published systems.

Paper	Model	Outcome	Training database	Evaluation database	AUC (IC 95%)
Gatellier et al. (2020)	Logistic regression	pH < 7.1	CTU-UHB	CTU-UHB (subset of 439 cases)	0.72 (*)
Abry et al. (2018)	Sparse support vector machine	pH < 7.05	Lyon	CTU-UHB (subset of 472 cases)	0.72 (*)
Petrozziello et al. (2018)	Convolutional neural network	pH < 7.05	Oxford	CTU-UHB	0.82 (*)
Ogasawara et al. (2021)	Convolutional neural network	pH < 7.2 or Apgar 1’ < 7	CTU-UHB	CTU-UHB	0.73 (0.69–0.77)
Fergus et al. (2021)	Convolutional neural network	Caesarean	CTU-UHB	CTU-UHB (subset of 101 cases)	0.73 (*)
**DeepCTG® 1.0**	**Logistic regression**	**pH < 7.05**	**SPaM, Beaujon**	**CTU-UHB**	**0.74 (0.66–0.81)**

* The 95% confidence intervals were not available for those studies.

Our model achieves a slightly better AUC than the 2 other models based on a logistic regression or a support vector machine (AUC = 0.74 vs. AUC = 0.72 or 0.73). The only model giving a better performance is the one published by Petrozziello et al. (26), which is trained on a much larger dataset with more than 35.000 cases and using deep learning methods. However, those differences in accuracy are very probably not statistically significant because of the important width of the confidence intervals due to the relatively small size of the CTU-UHB dataset.

### 3.6. Comparison of the model with expert obstetricians' annotations

**Figure 3:**
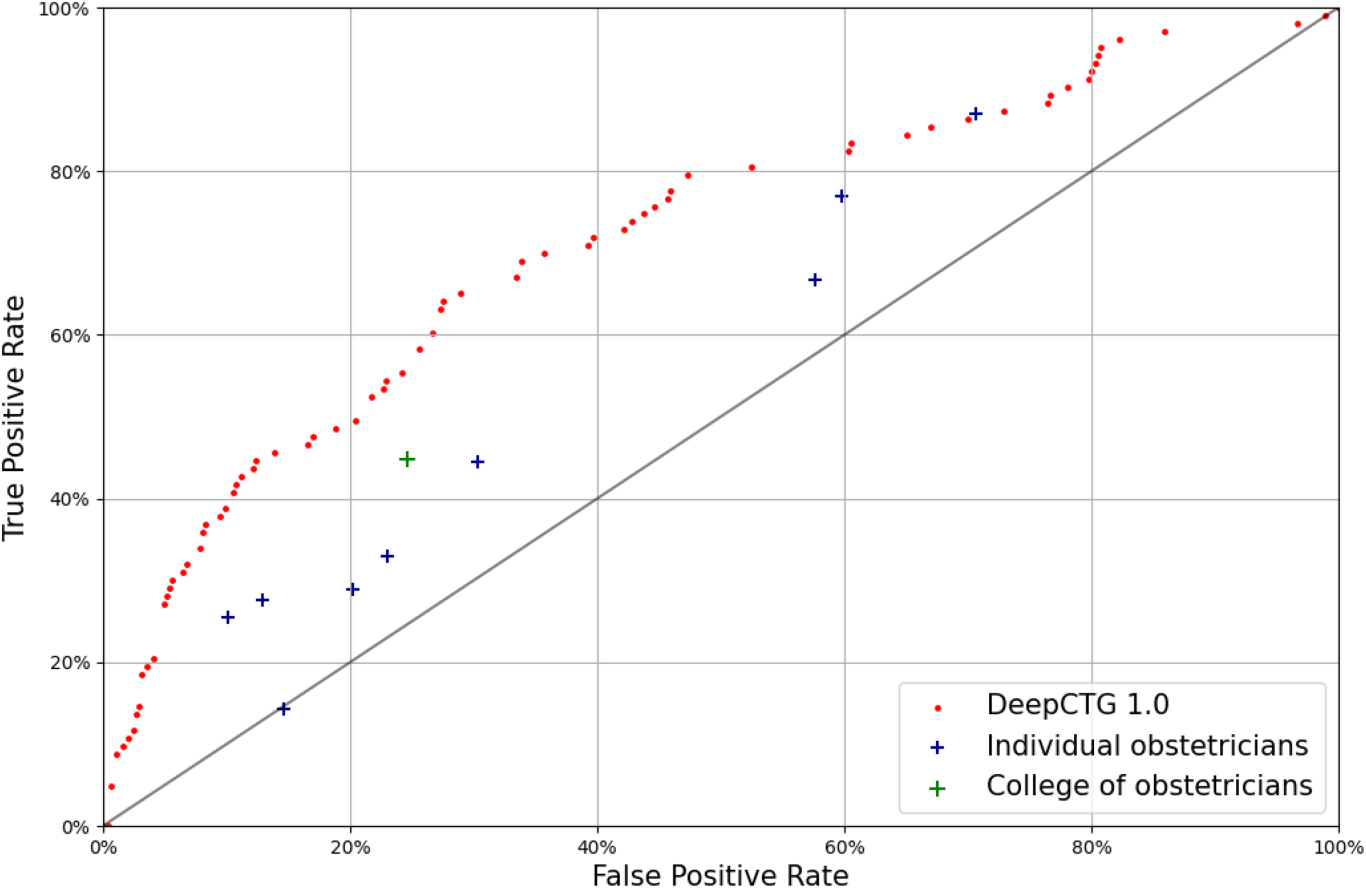
Receiver-operating characteristic curve of DeepCTG® 1.0 (red dots), compared with the obstetricians’ annotations of the CTU-UHB cases (outcome: pH < 7.15).

We have compared the performance of our model with the available obstetricians' annotations (Figure 3). The model is evaluated on the CTU-UHB dataset and trained on the other datasets with an outcome consistent with the annotations (pH < 7.15). The annotations differ significantly from an obstetrician to the other, confirming the important interobserver variability reported in past studies (5, 35).

The outcome predictions from the college of obstetricians are quite conservative, with a low false positive rate around 25%, but a low true positive rate around 45%. The model performs better than the college of obstetricians and better than every individual expert.

### 3.7. Impact of the inclusion of cesareans in the training and evaluation datasets

The mode of delivery (natural, operative or caesarean) is available for every birth in CTU-UHB and Beaujon datasets (Table 7). The rate of births with pH < 7.05 is much higher for cesareans (16% vs. 6% for other delivery modes). While the inclusion of cesareans in the training dataset does not impact the results significantly, their evaluation on cesareans only cases leads to a slightly worse performance (AUC = 0.74 vs. 0.76) (Table 8).

**Table 7:** Number of cases per delivery mode (CTU-UHB and beaujon datasets).

	Number of cases	Pathological cases (pH < 7.05): Number (%)
Cesareans	127	20 (16%)
Other delivery modes	1,100	62 (6%)

**Table 8:** AUC of the models depending on delivery modes included in training and evaluation datasets (CTU-UHB and beaujon datasets, k-fold cross-validation).

		Evaluation dataset	
		**Cesareans**	**Other delivery modes**
Training dataset	All delivery modes	0.735	0.758
	Without cesareans	0.735	0.760

### 3.8. Impact of the length of the CTG segment

The models presented up to this section are fed with features computed from 30 min CTG segments. We study here how the length of the CTG segment impacts the performance of the model. The AUC is 0.68, 0.76 and 0.78 with 10 min, 30 min and 60 min respectively (Table 9).

**Table 9:** Evaluation of DeepCTG® 1.0 depending on the length of the CTG segment fed to the system (all datasets, k-fold cross-validation).

Length of the CTG segment	AUC
10 min	0.679
20 min	0.747
30 min	0.760
40 min	0.773
50 min	0.774
60 min	0.779
75 min	0.788

## 4. Discussion

In this paper, we have introduced DeepCTG® 1.0, a model for predicting fetal hypoxia from the CTG signals. We have brought a significant attention to the methodology used to preprocess the CTG signals and to extract the features which are fed to the model. The methodology applied to fill the missing points in the signals did not have a significant impact on the model's performance, as mentioned in other studies (34), and we have used a simple approach based on linear interpolation. The most complex part is the computation of the FHR baseline, which is also a prerequisite for the computation of the features based on accelerations and decelerations. The baseline estimation methodology aims at reproducing as good as possible the annotations of baselines, accelerations and decelerations provided by a consensus of obstetricians in the FHRMA dataset. Our approach is based on a weighted median filter similar to the one introduced by Boudet et al. (30), which is shown to work better than other published approaches (29), and we have added a modulation of the duration of the window used to compute the baseline. An area for improvement in the preprocessing of the signals is to identify the maternal heart rate which is sometimes recorded by mistake by the monitor. Boudet et al. highlight the importance of this step when preprocessing the FHR signal (26).

The four features fed to the model (minimum and maximum value of the baseline, and the area covered by the accelerations and decelerations computed on a 30 min segment) have been selected among a larger set of 25 features. We have verified that using those 4 features only did not harm the performance of the model with the important advantage of keeping it simpler, confirming the results of past studies (19). This gives our model the important characteristic of being interpretable: it is based on features which are known and understood by practitioners, and the influence of each feature on every prediction can be evaluated.

While being relatively simple, it performs well on the three datasets used in this study. Very importantly, the performance on each dataset is evaluated when the model is trained on the remaining datasets, making it representative of the performance the model could reach when deployed in new centers without any specific adaptation. The performance obtained on the Beaujon dataset (AUC = 0.74) is representative of the performance that could be achieved in a clinical context, because it includes all pathological cases occurring at Beaujon hospital over a time period and non-pathological cases have been filtered for practical reasons, but the filtering methodology described previously should not bring any bias in the dataset. We do not know how the 552 cases in the open CTU-UHB dataset have been selected, but the performance of the model is very close on those cases. The performance is significantly higher on the SPaM dataset (between 0.77 and 0.87). This dataset has been built as part of a challenge and the cases may have been carefully selected, as the lower share of missing points in the FHR and UC signals in the SPaM dataset suggests. Also, the high difference in fetal pH between the pathological cases (pH < 7.05) and the non-pathological ones (pH in the range 7.25–7.30) very probably helps the model to classify the cases. This explains the higher performance observed on those cases.

We have compared the performance of the model with other published models and with obstetricians' annotations. The comparison with other models published is not straightforward, because the models are often evaluated on a different set of clinical cases or using different outcomes than pH < 7.05. Despite those limitations, we conclude that our model performs slightly better than other models based on relatively simple statistical models (like logistic regression or support vector machines) trained on datasets of similar sizes. The only model performing significantly better is the one published by Petrozziello et al. (26), which is based on a more complex deep learning model trained on a much larger dataset with more than 35.000 cases. Its complexity makes it harder to deploy in clinical practice and to provide interpretable indicators for practitioners. However, it is worth noting that those differences in accuracy are very probably not statistically significant because of the relatively small size of the CTU-UHB dataset. When compared with the annotations of the CTU-UHB cases by nine expert obstetricians, the model performs significantly better than every individual expert and the majority vote.

We have challenged the inclusion of cesareans in the datasets used to build the model, as this is a questionable decision. On one hand, for those births, there is probably a lower correlation between the available CTG signals and the fetal pH at birth. Indeed, there may be a delay between the end of the CTG segment and the time of delivery, as in practice the CTG signal does not end at delivery but instead when the decision of doing the cesarean is made. On the other hand, removing those cases could create a very strong selection bias harming the performance of the model when it is used in clinical practice. As far as we know, this point is not discussed specifically in the literature and more research should be done to integrate those births consistently when building the model by using the reason why the cesarean was performed. Our study shows that the inclusion of cesareans in the training dataset does not significantly impact the accuracy of the system, suggesting that for most cesarean cases, the last available CTG signal can be reliably used as a proxy of the state of the fetus just after delivery.

The definition of the CTG segment used to compute the features is a key parameter of the system: in this study, we choose the last 30 min before delivery. We have shown a high dependence of the performance of the system on the length of the segment: we choose 30 min, which achieves a good performance while making the model usable quickly after the CTG recording starts. Using the latest available signal before delivery is consistent with the fact that the outcome is based on the fetal blood pH measured right after delivery. However, the signal during the expulsive period is probably more difficult to interpret. Future research will aim at using a fetal outcome based on fetal blood sampling during labor, removing the need to use the signal during the expulsive period to train and evaluate the system. Also, in a clinical setting, the system would be applied to detect fetal hypoxia as early as possible to help appropriate intervention. Further research will aim at optimizing the system to improve early detection of fetal hypoxia, for example by using the signal earlier in the labor to predict the fetal outcome. This point is not specifically studied in the literature as far as we know.

In this work, cases of fetal hypoxia are defined by a fetal pH at birth lower than 7.05, which is the most common choice in the literature. Using a higher threshold may be more relevant in clinical practice to detect fetal hypoxia as soon as possible and to use the model as a non-invasive second line to replace fetal blood sample. Using other fetal outcomes not based only on the fetal pH can be considered (36), as a low pH alone is not synonymous with a poor neonatal condition (37). Other computerized systems use outcomes based on the Apgar score (21) or on both pH and Apgar score (23).

We identify several areas for improvements that will be the focus of future work. A major improvement is to integrate the clinical context in the model, as done in a few studies using for example preeclampsia and thick meconium, nulliparity or the labor stage (15, 38, 39). Also, the use of more advanced machine learning or deep learning algorithms could help to model the complex correlations between the CTG signals, the clinical context and the fetal outcome, at the price of a more complex and less interpretable model. Finally, training and evaluating the model on larger databases with more pathological cases and covering more maternity centers should help to improve the performance and robustness of the model.

## Data Availability

The raw data supporting the conclusions of this article will be made available by the authors, without undue reservation.
